# A process evaluation of the quality improvement collaborative for a community-based family planning learning site in Uganda

**DOI:** 10.12688/gatesopenres.12973.2

**Published:** 2019-08-29

**Authors:** Christine Kim, Ramadhan Kirunda, Frederick Mubiru, Nilufar Rakhmanova, Leigh Wynne

**Affiliations:** 1 Health Policy and Management, Gillings School of Global Public Health, University of North Carolina at Chapel Hill, 135 Dauer Drive, Chapel Hill, NC, 27599-7411, USA; 2 FHI 360, Kitante Close, Kampala, Uganda; 3 FHI 360, 825 Connecticut Ave NW, Washington, DC, 20009, USA; 4 FHI 360, 359 Blackwell Street #200, Durham, NC, 27701, USA

**Keywords:** quality improvement, improvement collaborative, community-based family planning, community health workers

## Abstract

**Background**: High-quality family planning (FP) services have been associated with increased FP service demand and use, resulting in improved health outcomes for women. Community-based family planning (CBFP) is a key strategy in expanding access to FP services through community health workers or Village Health Team (VHTs) members in Uganda. We established the first CBFP learning site in Busia district, Uganda, using a quality improvement collaborative (QIC) model. This process evaluation aims to understand the QIC adaptation process, supportive implementation factors and trends in FP uptake and retention.

**Methods:** We collected data from two program districts: Busia (learning site) and Oyam (scale-up). We used a descriptive mixed-methods process evaluation design: desk review of program documents, program monitoring data and in-depth interviews and focus group discussions.

**Results:** The quality improvement (QI) process strengthened linkages between health services provided in communities and health centers. Routine interaction of VHTs, clients and midwives generated improvement ideas. Participants reported increased learning through midwife mentorship of VHTs, supportive supervision, monthly meetings, data interpretation and learning sessions. Three areas for potential sustainability and institutionalization of the QI efforts were identified: the integration of QI into other services, district-level plans and support for the QIC and motivation of QI teams. Challenges in the replication of this model include the community-level capacity for data recording and interpretation, the need to simplify QI terminology and tools for VHTs and travel reimbursements for meetings. We found positive trends in the number of women on an FP method, the number of returning clients and the number of couples counseled.

**Conclusions:** A QIC can be a positive approach to improve VHT service delivery. Working with VHTs on QI presents specific challenges compared to working at the facility level. To strengthen the implementation of this CBFP QIC and other community-based QICs, we provide program-relevant recommendations.

## Abbreviations

APC, Advancing Partners & Communities Project; CBFP, Community-based Family Planning; CHW, Community Health Worker; FGD, Focus group discussion; FP, Family Planning; HMIS, Health Management Information System; IDI, In-depth interview; LS, Learning Session; MOH, Ministry of Health; PDSA, Plan-Do-Study-Act; VHT, Village Health Teams; QI, quality improvement; QIC, Quality Improvement Collaborative

## Introduction

**Figure 1.**
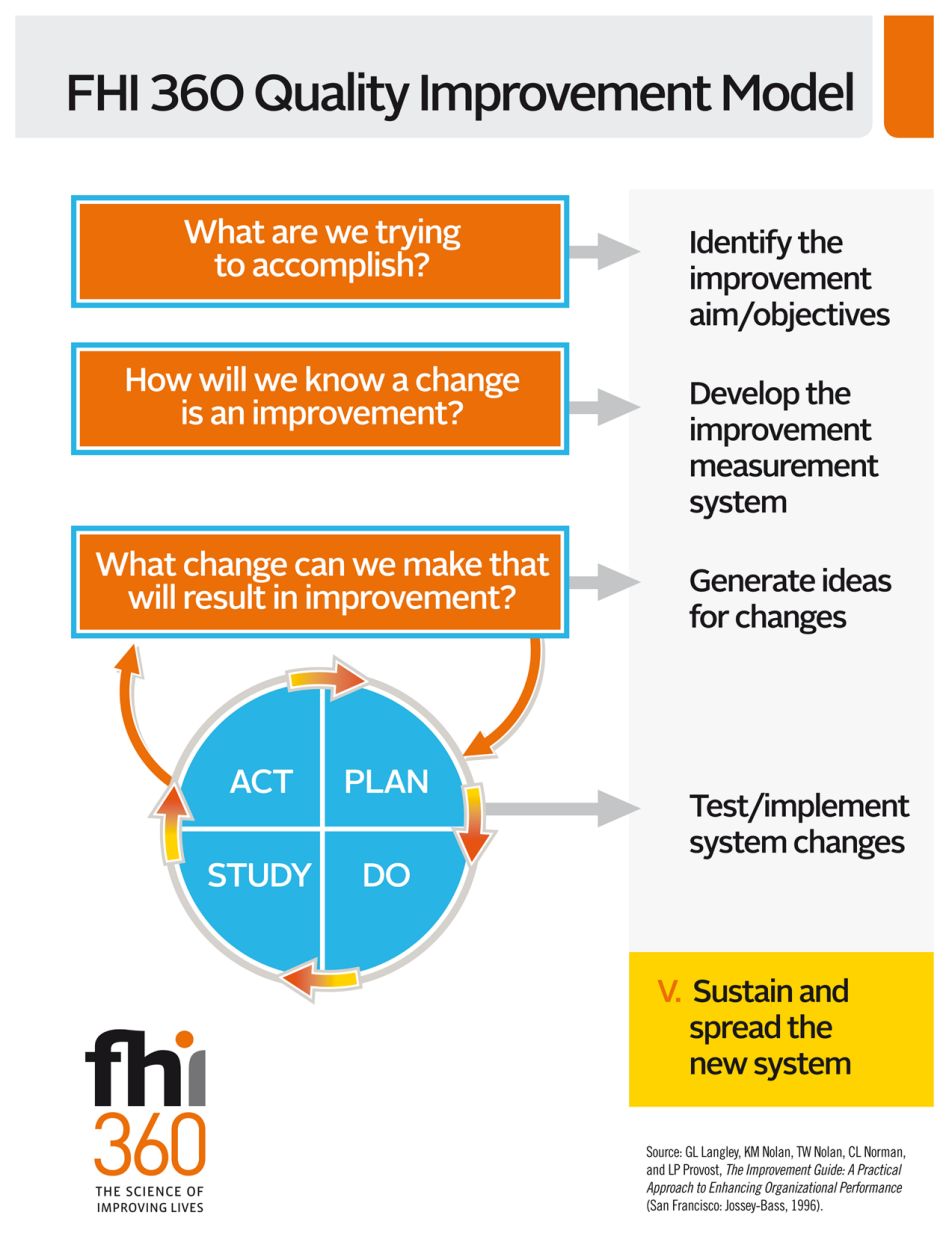
FHI 360 Quality Improvement Model.

The integration of volunteer community health workers (CHWs), or Village Health Team (VHT) members in Uganda, into family planning (FP) service delivery is one of several proven high-impact practices in family planning and is called community-based family planning (CBFP) ^1^. A review of CBFP programs over the last three decades found that community distribution of FP was necessary in country contexts where clinics and hospitals could not meet the FP needs of communities ^2^. High-quality FP services have been associated with increased FP service demand and use, resulting in improved health outcomes for women, including reductions in unmet need for FP and rates of unintended pregnancy, abortion, morbidity and mortality ^3^. Quality improvement collaboratives (QIC) have been adopted widely as an effective team-based learning and improvement approach ^4^. QICs have been implemented across sub-Saharan Africa, focusing on iteratively testing changes in service delivery and analyzing their effects on processes and outcomes ^5, 6^, often using the plan-do-study-act (PDSA) quality improvement model ( Figure 1).

These quality improvement initiatives are often clinical and disease-focused (e.g., HIV/AIDS), driven by clinical processes (e.g., appropriate treatment, diagnosis) and have occurred at the health center level ^7, 8^. Evaluations of quality improvement initiatives in Sub-Saharan Africa are limited, with examples from Uganda and Tanzania on maternal and newborn healthcare ^6, 9^ and from northern Ghana on maternal and child health ^10^.

In Uganda, the total unmet need for FP is 28.0% ^11^. This unmet need is highest among the two poorest wealth quintiles and among women 25 to 35 years of age ^11^. Among current female users of FP, only 61.7% were counseled on side effects and 60.8% were told of other methods ^11^. To our knowledge, there is no documentation in the published literature or the Ugandan national health strategies of QICs targeting CBFP service delivery models.

While a variety of QICs have been used in healthcare, it is often unclear what makes them successes or failures ^12– 14^. Process evaluations are particularly useful tools in the QIC context, because they allow for detailed understanding of activities actually performed and participants’ experiences, which informs revision and adaptation of the intervention— a natural and iterative process within the QIC model ^15, 16^. The Bruce Framework includes six elements that are essential for high quality FP services: 1) choice of methods, 2) information provided to clients, 3) service provider competence, 4) interpersonal relations, 5) continuity and follow-up mechanisms, and 6) appropriate constellation of services ^17^.

This process evaluation was conducted in a QIC’s learning site in Busia district and one of the scale-up districts, Oyam. We analyzed the trends in FP uptake and client retention by VHTs and aimed to identify the factors that were supportive of the CBFP QIC implementation, as perceived by the collaborative actors and in relation to the Bruce Framework.

## The QIC

In 2014, Uganda’s Ministry of Health (MOH) selected Busia district to serve as a CBFP learning site for Uganda. Three health centers with established CBFP programs comprised the QIC learning site. A more detailed description of the QIC we evaluated is provided elsewhere ^18, 19^.

**Figure 2.**
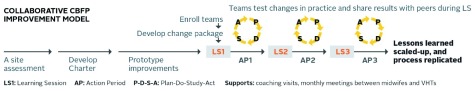
Collaborative Improvement Model.

The first step of the QIC (see Figure 2) was to conduct a collaborative site assessment, which looked at the overall CBFP system, training, supervision, referrals, supplies, infection prevention, waste management and reporting at the district, health center, community health worker and client levels in the learning site’s catchment area. Upon completion, a dissemination meeting was held to achieve understanding of the strengths and areas for improvement, as well as potential solutions to be included in a QI Charter, which would state the aim of the improvement effort and provide a roadmap for the QIC. Subsequently, in collaboration with clients and other local stakeholders, the collaborative members (e.g., midwives, in-charges of health facilities (usually physicians), VHTs and district health officials) developed, implemented and tested innovative change ideas using the PDSA method.

Approximately every six months, learning sessions were convened for collaborative members to share with and learn from peers who had successfully implemented or replicated change ideas. The first learning session (LS1) was internal, for collaborative members of the learning site (Busia) and a few new health centers in that district that were preparing for scale-up. For LS2, a delegation from the scale-up district (Oyam) came to the learning site to take learning back to their district. LS3 took place in Oyam district with a delegation from the learning site and two additional districts where the collaborative was being scaled-up. This process evaluation was completed before LS3 and subsequent scale-up plans to other districts.

For each LS, the agenda was tailored to the dynamics and the context of each group. This process of adapting the learning sessions with new materials and new roles for participants was designed to continually challenge the QI teams and enhance Busia’s role as a learning site. In addition, clients were engaged throughout the QIC and helped generate change ideas to ensure a client-centered approach. The QIC aligned with all five objectives of the MOH’s Health Sector Quality Improvement Framework and Strategic Plan (2015/2016-2019/2020) ^20^.

## Methods

We used a descriptive mixed-methods process evaluation design, including the following components: desk review of program documents, extraction of program monitoring data, and qualitative research methods. We collected data from two program districts: Busia (learning site) and Oyam (scale-up).

### Study setting

Busia district is in the eastern region of Uganda. Busia has been implementing CBFP since 2008. As a border district with Kenya, Busia is densely populated, with both rural and peri-urban areas, has one of the highest adolescent pregnancy rates in Uganda and a total fertility rate of 6.0. Oyam district is a relatively new rural district in the northern part of Uganda, with a total fertility rate of 6.3, which is higher than the national average of 5.4 ^21^. CBFP was introduced to Oyam in 2015.

### Sample population

The target populations for this study were the core members of the QI teams: VHTs, midwives and their clients. QI teams are composed of midwives, district health managers, health center in-charges and VHTs. QI team members from Busia were eligible if they had been a member of the QI team at their health center and had attended at least one learning session, followed by implementation of at least one PDSA cycle. To select participants for focus group discussions (FGDs) and in-depth interviews (IDIs), we purposively identified a subset of pilot and scale-up health centers in Busia and Oyam districts. VHTs were short-listed by health center based on eligibility criteria and selected using a random number generator from the list for up to 7 to 10 VHTs per health center. 

Using a random number generator, we randomly selected male and female clients at least 18 years of age for FGDs and couples for IDIs who were recorded in the VHT registers during the implementation period (June 2015-December 2016). IDIs were conducted with one midwife and in-charge from each health center. All but two of the health centers in Busia had only one midwife and one in-charge. The midwife responsible for supervision and coaching of VHTs providing CBFP was interviewed at the health center with more than one midwife. Representatives from the MOH at the district and central levels who were familiar with the CBFP QIC were interviewed. All participants located in the two districts were contacted by mobile phone and invited to participate. The MOH representative at the central level was contacted by email. We also sent an email invitation to an NGO representative from a health facility QI program who did not respond. No invited participants refused to participate or dropped out. The total sample size for each participant group is listed in Table 1.

**Table 1.** Sample of focus group discussion (FGD) and interview participants.

Participant group	Total, N	Busia, n	Oyam, n	FGD, n	IDI, n
VHTs	76	52	24	10	-
Midwives	7	5	2	-	6
In-charge	5	4	1	-	5
District officials	5	2	3	-	5
Ministry official	1	-	-	-	1
Clients	35	35	-	-	-
Couples	4	4	-	-	4
Females	19	19	-	2	-
Males	8	8	-	1	-
Total	129	98	30	13	21

IDI, in-depth interview; VHT, Village Health Team.

### Data collection

***Review of documents.*** We reviewed program documentation to understand the processes and evolution of the QIC that was implemented by health centers in Busia district from March 2015 to May 2017 and in Oyam district from July 2016 to May 2017. We identified what change ideas were implemented over time and challenges reported in order to understand the context of the trends shown in the data. These documents included: collaborative site assessment reports at baseline, QI indicator data, routine client satisfaction surveys and coaching visit reports, district charters, trip reports, learning briefs, and other program documents.

***Quantitative data.*** We tracked the following three indicators using information from the VHT client registers collected by the program from January 2015 to March 2017: number of female clients on FP returning to the VHTs, total number of VHTs’ female clients on FP, and number of couples counseled on FP by the VHTs. All data were de-identified, then aggregated by health facility and month.

***Qualitative data.*** Local research assistants conducted IDIs and FGDs with clients and VHTs in each participant’s language of preference: Luganda, Samia, Ateso, Langi or English. One interviewer conducted IDIs and a team of two (one facilitator and one note taker) conducted the FGDs. All interviews with midwives, in-charges and government officials were done in English. Interviews were digitally voice recorded upon receipt of each participant’s informed consent to proceed with the interview, field notes, and the voice recordings. Each IDI took approximately 30 minutes, and each FGD took no longer than 45 minutes (per Ugandan IRB requirements). Interviews and discussions were conducted using a semi-structured guide covering topics relevant for the target participant group, such as key features of QI, what worked, challenges, the adaptation process, sustainability and institutionalization and perceptions of the quality of services (see *Extended data* ^22^). We conducted interviews over a two-week period in May 2017. We aimed to reach apriority thematic saturation and data saturation as described by Saunders *et al*. ^23^. All interviews and discussions were conducted at a quiet and private location at the health centers. All audio records of interviews were transcribed into English and de-identified.

### Data analysis

We cleaned the quantitative data using Stata 14 and collapsed each indicator into monthly sums by district ^24^. We obtained descriptive statistics (counts and means per indicator) and plotted the data on run charts in Microsoft Excel. Run charts are simple to construct and are a useful tool for learning about the performance of a QIC. Our run charts demonstrate over a 28-month period whether we have non-random patterns (“signals”), using the median of the total data points collected during that timeframe as a centerline. The median, which indicates the point at which half the data are either above or below the line, is not influenced by extreme values. In this process evaluation, the question we sought to answer was not whether any changes observed were statistically significant, but to determine whether they represented sustainable improvement over time. To do so, we used runs charts to identify trends and shifts ^25, 26^.

We used NVivo 11 to conduct a content analysis of the qualitative data transcripts ^27^ and developed an integrated approach to coding ^28^. After reviewing all the transcripts to gain an understanding of and context for the data, we developed a coding structure deductively based on the previous review of program documents and overall review of the data. Two researchers (CK and RK) worked independently to code the transcripts. After initially coding a few transcripts, the two coders discussed their approaches and agreed upon a coding structure of major themes. Inter-coder reliability and agreement were checked by comparing the analyses of the two coders, assessing the overall level of agreed codes, and discussing areas of disagreement. We used NVivo’s querying capabilities to assess the frequency of codes, assessing codes by attributes and co-occurring thematic codes. We extracted illustrative quotes for each major theme.

### Ethical considerations

Participants were informed about the aim of the research project and gave consent to participate. The District Health Quality Improvement Officers leading the health operations in the two districts were part of the team and approved the publication. During the informed consent process, respondents were informed that any information we include in reports would not identify them. Participants did not review the data and provide feedback.

All study documents were reviewed, approved, and categorized as exempt from research status, as it was deemed as posing no more than minimal risk, by FHI 360’s Protection of Human Subjects Committee (Project #: 1035307), the Research Ethics Committee of The AIDS Support Organization in Uganda (Ref. #: TASOREC/15/17-UG-REC-009) and the Uganda National Council for Science and Technology (Protocol #: SS 4267). District offices and health facilities were informed of data collection plans prior to interviews and focus group discussions through phone calls and emails by APC staff. Verbal informed consent was obtained from all interview and focus group discussion participants to minimize links between participants, other people’s knowledge of any participants’ participation in the study, and information they provide. All participants were given paper copies of the informed consent with information on whom to contact with further questions in either English or a local language of their choice.

## Results

### Trends in key indicators

**Figure 3.**
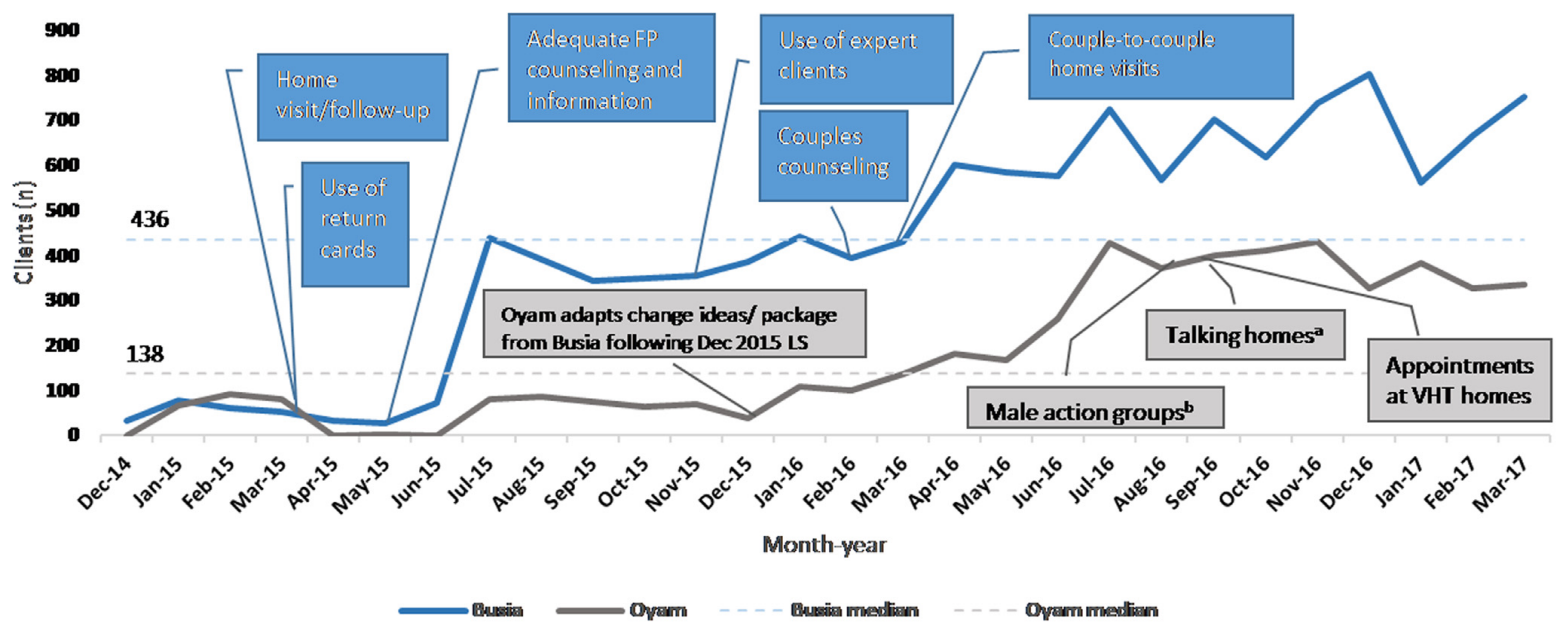
Number of family planning (FP) clients returning for follow-up, December 2014 - March 2017. Annotated run chart with examples of change ideas implemented over time. ^a^Talking homes: displaying FP posters and providing confidential space in Village Health Team (VHT) home for services. ^b^Male action groups: groups of men formed to support community mobilization and dissemination efforts typically found in Northern Uganda. These men were selected by community members based on trust and respect.

**Figure 4.**
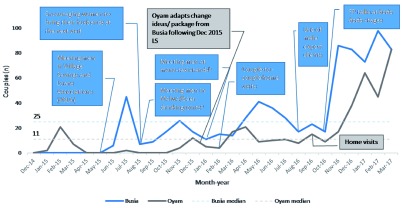
Number of couples counseled, December 2014- March 2017. Annotated run chart with examples of change ideas implemented over time. ^a^Meeting men in Malwa/beer drinking joints: Village Health Team (VHT) members meet with men at popular drinking joints to talk to them about family planning (FP) services using their elevator speeches. ^b^Meeting men at moto-taxi stands: VHTs meet with male motorbike taxi drivers at their waiting stands to talk to them about FP services using their elevator speeches.

There was an increase in the number of female FP clients returning to a VHT for follow-up and resupply in Busia and Oyam districts from December 2014 to March 2017 ( Figure 3). In Busia, a positive trend (defined as five data points in one direction ^26^) is shown from March 2016 to March 2017, with 14 data points above the median line of 436 clients. Oyam shows a similarly positive trend, with 12 data points above the median line of 138 clients. These positive trends occurred as several changes were being introduced and tested, including home visits by VHTs, use of expert clients, and couples visiting other couples. A similar trend was seen in the number of female FP clients, with a median of 694 for Busia and 228 for Oyam (see *Underlying data* ^29^). Successfully engaging men was one of the greatest challenges. However, after targeted sensitization and education efforts by VHTs to specifically engage men, we saw dramatic improvements well above the median (25 couples in Busia, 11 couples in Oyam) in the number of couples counseled by VHTs starting in October 2016 ( Figure 4).

### Characteristics of respondents

We interviewed six key informant groups across the community, health center, district and central levels. Table 2 shows the average age of the VHTs who participated in the FGDs was 44.2 years, with an average of 12.7 years of experience as VHT, of which 3.8 years included CBFP experience. The average number of clients per month for VHTs in both districts was 14.7. The Busia VHTs had more years of experience overall (13.8 years vs. 10.8 years) and in CBFP (4.9 years vs. 1.8 years) compared to those in Oyam. Midwives had an average of 7.9 years of work experience, but three out of the four midwives interviewed had been at their designated health centers for less than a year, indicating the endemic challenge of staff turnover. Clients’ average number of years on an FP method was 2.6 years, with the most commonly reported method choice being an injectable (e.g., DMPA-IM, DMPA-SC).

**Table 2.** Characteristics of respondents.

Variable	Mean age, years	Mean years of work	Mean VHTs supervised, n	Mean years of CBFP	Mean clients, n
VHTs	44.2	12.7		3.8	14.7
Busia		13.8		4.9	16.1
Oyam		10.8		1.8	11.6
Midwives		7.9	16.9		141.7
In-charge		8.6			
District officials		4.5			
Ministry official		6			
	Mean age, years	Mean age of husband, years	Mean children, n	Mean ideal no. of children, n	Mean time on FP, years
Clients	34.7		4.7	5.6	2.6
Female	31.0	36.9		5.2	
Male	42.5			5.9	

VHT, Village Health Team.

### Learning site’s experience with a QIC

The key features of the QIC in Busia were the application of the PDSA model to test and implement change ideas, supervision and mentorship of VHTs by midwives, monthly meetings and collection and use of data by VHTs. Factors supporting QIC implementation include all factors that emphasized or maximized making changes in VHTs’ practices or their learning. There were differences in how the PDSA was described by participants, particularly between the VHTs and higher-level staff (e.g., midwives, in-charges, district QI focal persons and district health officers). Higher-level staff referred explicitly to “PDSA,” while most VHTs and some midwives described the process of looking at the data, assessing where there are gaps and finding solutions to improve performance. VHTs focused on specific change ideas that they had implemented as part of their CBFP routines. Participants in every higher-level cadre noted being surprised that VHTs could learn to use the PDSA model, which had mainly been used by health workers for clinical processes:

*“With the VHTs it was very interesting that in the beginning, I could not believe that they could apply the PDSA […] it was very important because […] after identifying specifically what their communities are, they could identify their change ideas, go try them, then give feedback themselves. They have applied and then they say this one has worked out, we want to continue with it. […] Others say they are not working, they have modified them, others they have abandoned.” –* Busia district official

The district official saw that the PDSA process allows different communities to adapt ideas to their contexts, test them on a small scale, and then abandon or adapt and scale them up. The use of data and visuals, such as run charts ( Figure 3, Figure 4), to track progress gave VHTs a stronger evidence base for deciding whether to retain, modify or abandon their change ideas:

*“To know that we have worked hard, we look at our run chart to see the indicators — whether we have dropped or increased. If we dropped, we look at our change ideas and modify them to see a change from dropping to increasing for the next month.” – Busia VHT*

Participants also mentioned the value of having more structured monthly meetings and the importance of supervision and mentoring by midwives for improving quality. All midwives reported meeting monthly with the VHTs prior to the QIC, but only so the VHTs could submit their monthly health management information system reports. These meetings are now being used to discuss achievements and challenges, study the data, learn and encourage one another. Responses indicate that quality improvement activities can be integrated into existing meetings:

*“These monthly meetings started before the QI approach anyway, but with the QI approach, it has helped because there were certain areas that before we were not covering. But from the time we were implementing QI, we improved on those areas. We realized that the VHTs were not giving enough information on family planning, as in they were not giving information on all available methods, but only ones they are giving. But now they are able to give all those information.” – Oyam midwife*
*“Our coming here [to the health center] monthly has helped us to know what challenges our fellow VHTs are facing in their communities and what challenges I am facing, and when we sit and discuss, we come up with solutions to those challenges.” – Busia VHT*

The changes the VHTs considered most effective were adequate counseling with job aids, home visits/client follow-up, sensitization and mobilization and male involvement activities, described in Table 3.

**Table 3.** Change ideas considered most effective by Village Health Teams.

Change idea	Description
Adequate counseling with job aids	Fully counseling clients on all family planning methods with the use of pictorial job aids, including counseling on side effects
Home visits and client follow-up	Visiting clients in their homes for counseling, particularly for reaching couples; following up with clients by phone and with appointment cards for resupply
Sensitization and mobilization	Sensitizing target community members through different approaches **Target groups:** *boda boda* (motorcycle transportation) stages for men, church meetings, women’s savings groups, beer joints **Methods:** “elevator speeches” and expert clients
Male involvement activities	Engaging men with FP information, specifically at *boda boda* (motorcycle transportation) stages and beer joints, increased couples counseling

FP, family planning.

Factors cited as contributing to the maintenance and sustainability of QIC were the inclusion of local leaders in the first LS in Busia (which included training with guidance materials/job aids) and ensuring client confidentiality. By engaging clients in the assessment and each LS throughout the QIC, VHTs became more aware of the importance of trust and confidentiality.

“ *I am trustworthy with the secrets of clients. When I counsel a client I never move around disclosing to other people what they have told me. So, I am sure those clients talk to their friends about my services and refer them to me.” – Busia VHT*

### Experience with the QIC in scale-up

During a joint LS in Busia, Oyam midwives and VHTs were paired with their counterparts from Busia. The Busia midwives and VHTs shared their QIC experiences, including what worked and their implementation of the QIC. The lessons from that experience most commonly mentioned by Oyam respondents in the FGDs and IDIs were mentorship by midwives, data collection and use, infection prevention and using the PDSA process.

Busia midwives shared their client registers, run charts, mentorship schedules, approaches to monthly meetings, use of a supervision checklist, and how they delegated responsibilities to higher-capacity VHTs at their health centers (i.e., compiling monthly VHT reports) to reduce workloads.

*“From Busia I learned that the other midwife was having monthly meetings, and another thing she was doing … mentoring. Now I always sit down with them at the end of each week to see what they are not performing and what each one is not doing well, and I mentor.” – Oyam midwife*
“ *Her [Busia midwife’s] ideas helped me a lot. I used to do data collection alone. But now my VHTs will make the summary. That has really helped me a lot, because I was even breaking. But now, I feel relieved.” – Oyam midwife*

Busia VHTs showed the Oyam VHTs their VHT client registers and how they entered data. They also discussed how they conducted adequate counseling with the job aids, their change ideas and building relationships with their clients. Oyam participants then visited a VHT’s home to observe how privacy was achieved, storage of supplies and infection prevention. Observing how Busia VHTs maintained infection prevention seemed to make a lasting impression on Oyam VHTs:

*“Before we had gone to Busia, after administering the drug to the clients we disposed of it not in the way we were supposed to […] but after we went to Busia we learnt that we should dispose them [cotton wool, needles] off separately.” – Oyam VHT*
*“They also gave information to prepare a point for washing hands, one at the entrance of latrines and another near the place where I administer medication to my clients.” – Oyam VHT*

When they started implementing the QIC, the Oyam team members found that some of what they had learned from Busia needed to be adapted to the Oyam context:

*“We went to Busia and learned much about the PDSA cycle, where we also identified our own problems, our own weakness, our own areas where we are not performing well, and we’ll bring a change idea. I also think what worked well in Busia may also work well with us, but we believe that communities vary. We don’t have the same cultural practices and beliefs, because it is the culture and belief of the community that determines their uptake of the services that we offer to them. So, we also want to reserve our own ideas. But what we learned from them is how to go on with the PDSA cycle. Even more new ideas will work.” – Oyam district official*

Oyam VHTs and midwives were inspired by many good practices in Busia but had the freedom to adapt those ideas. They also showed that effective mentoring is possible with simple supervision tools and practices, such as checklists and spot checks. They reported frequent peer-to-peer learning between VHTs and midwifes. Overall, their responses indicate that well designed learning exchanges are a critical step in the scale-up process.

### Perceived effects of QIC

The greatest perceived direct effects of the QIC were related to improved client satisfaction and trust, changes in the ways the teams worked and improved quality of VHT services. All VHTs and midwives reported increased client satisfaction and trust in the CBFP services delivered by the VHTs due to the improved quality of those services, which they said had resulted in increased numbers of FP clients and better client retention. Midwives and VHTs both reported improvements in VHTs’ ability to counsel clients on side effects and refer them to the health center when necessary for long-acting and permanent methods, serious side effects and/or complications.

*“[Unnecessary] referrals were minimized because most of them were running to me, ‘ah, the VHT gave me depo, I’m bleeding, I’m feeling like this…’ those side effects, so when they emphasized critical counseling, we’re seeing less at the [health center] because they explain to the client, you are most likely to get such and such side effects, but if you get such and such side effects, or your body is not yet used so you might feel like this, so they came to understand that now.” – Busia midwife*
*“The work the VHTs have been doing is great, because if I get a complication on the method I am using for family planning, she would help us, because coming to the health center was a far distance and her being in the community helps us go to her for medication and saves us the cost of transport and medicine to use on time, because sometimes it finds you without any funds to come to the health center, so we call her when we need her.” – Busia client*

An emphasis on teamwork allowed for shifts in responsibility, freeing midwives to focus on complicated cases. Both VHTs and midwives described improved relationships with one another, describing how VHTs are more likely to seek the help of midwives and midwives take greater initiative in supporting and mentoring their VHTs through phone calls, spot checks at their homes and one-on-one supervision sessions.

Some indirect effects of the QIC were also reported by health center staff. These include changes in the overall health center’s QI efforts and integration of FP QI practices in other health service areas.

*“[The QIC is] more visible in maternity. Everything is well displayed, it’s a talking environment, people are talking about what is happening there. Most colleagues that go there are appreciating that [QI] is being practiced. So, I think [CBFP QIC] has spilled over […] they have made sure that other areas also get quality improvement.” – Busia in-charge*
“ *These days we integrate our work with the change ideas, because we are used to it and have been able to kill two birds with one stone […]so it is not limited to only FP.” – Busia VHT*

### Challenges to implementation of the QIC

Table 4 highlights the main challenges to implementation in each district. In Oyam, the primary challenges to implementation revolved around the complexity of the QIC in the beginning, but over time VHTs reported becoming more comfortable with the process. By comparison, Busia VHTs had fewer concerns about complexity and expressed greater confidence in their understanding of the indicators and data; they also had several more years’ experience implementing CBFP. The Busia VHTs’ main concerns were related to having a supportive environment to implement quality CBFP services. The differences in expressed challenges demonstrate an expected pattern in pilot (Busia) and scale-up (Oyam) QI teams. Pilot teams typically received more capacity building and support directly from QI specialists, as opposed to scale-up sites where the focus is on peer-to-peer learning. Therefore, the pilot teams were more familiar with the terminology and methods of measuring progress and were able to move to the next stage of discovering system challenges. 

**Table 4.** Main challenges.

Busia	Oyam
• Limited supportive environment: Unreliable stock and supplies, lack of transportation expenses • Engaging men in FP	• Complexity of QIC: data interpretation, indicators, run charts, implementation of change ideas • Increased workload • Transportation costs • Engaging men in FP

FP, family planning; QIC, quality improvement collaborative.

Participants from both districts mentioned the challenge of transportation costs and the limitations of the quarterly travel stipends provided by the project. The travel stipend was provided for VHTs to attend monthly meetings at health centers with the midwives. Some VHTs spend hours traveling from their villages. Many suggested that the stipends were not sufficient; VHTs incur additional costs when they conduct home visits in their large catchment area, and midwives incur costs conducting supervisory spot checks at VHTs’ homes. Frequently these costs are out-of-pocket expenses.

Another challenge was the availability of stock and supplies. As the number of FP clients increased, VHTs had difficulty projecting commodity requirements and would take either too much or too little. Finally, VHTs said that engaging men in FP was challenging, but necessary. VHTs reported not having enough time to counsel women adequately, because their husbands were not aware that they were using FP. Clients were often rushed and/or unable to return on time for fear of being caught by their husbands. VHTs also reported that men often believe in myths and misconceptions, are more difficult to meet during seasons of planting and harvesting when they work in the fields and can be abusive toward VHTs for introducing their wives to FP methods without their consent.

### Sustainability and institutionalization

Sustainability and institutionalization of the QIC, with Busia continuing to serve as a learning site, is essential for longer-term change in the delivery of quality FP services. Three areas arose in the interviews highlighting the potential sustainability and institutionalization of the QIC: 1) the integration of QI into other service areas; 2) district-level plans and support for QI in general and for CBFP; and 3) motivation of QI teams, especially VHTs and midwives. All health center staff and district officials from both districts expressed the utility of applying the PDSA approach to all health service areas and engaging VHTs in the process. Health workers reported integrating QI with other maternity services, immunization, HIV care and treatment, and health promotion and sanitation. A new project has been introduced specifically for QI in immunization, which has benefited from the training the VHTs had received. Busia proposed a budget for CBFP in its district costed implementation plan for FP. However, financing for QIC-specific activities centrally and at the district levels remains a challenge, and there are concerns about ensuring a continuous supply of FP commodities. Thus, while there is strong support and commitment for Busia to continue as a CBFP QIC learning site, sustaining it without committed government or project resources will be a challenge:

*“The idea of having a learning center or learning district is very important, especially in a resource limited setting. When you introduce something, people think it cannot work, but when you tell them that another district with the same environment has begun to do it, then they can think, okay, we can do it. So, it’s very important. That’s why we put it in the [national QI] framework.” –MOH official*
*“When [FHI 360] leaves us, it doesn’t mean they will collect the materials or knowledge that they have given us. The knowledge remains. I [am] happy about the mechanisms that they have given us, and will remain in us, for example, using the PDSA. As volunteers we can still come together, plan what we are going to do. […] The knowledge…shall maintain.”- Oyam VHT*

## Discussion

Global evidence has shown that CHWs are competent providers of CBFP services, including injectable contraceptives ^30– 32^. However, competence does not equate to quality health service provision. Greater efforts are needed to ensure that CHWs are continuously supported, mentored and monitored for improved service delivery to make effective gains in decreasing the unmet need for FP and increasing the access and use of modern contraceptive methods. This process evaluation shows that the QIC approach applied largely at the health facility level can be adapted and applied to community-based service delivery models.

Table 5 summarizes how the QIC approach supports and emphasizes the Bruce Framework components for quality FP services. QIC efforts to address the six elements of the Bruce Framework were not dependent on highly innovative or complicated change ideas. Rather, VHTs were provided a safe space to thoughtfully discuss their challenges and achievements, identify what they were doing right or incorrectly and support one another to try new ideas to achieve their improvement goals. The activities described are iterative and require time for learning and building trust in relationships among team members and patience for mentoring, with a long-term goal of continuous improvement through an institutionalized collaborative process.

**Table 5.** How collaborative improvement supports Bruce Framework components.

Bruce Framework components	Quality improvement elements that had positive effects
Choice of methods	• Use of job aids to guide counseling on all methods and side effects • Use of a supervisory checklist and spot check to improve supervision of VHTs by midwives • Peer-to-peer learning between midwives and VHTs, VHTs and VHTs
Information provided to clients	• Use of job aids to guide counseling on all methods and side effects • Emphasis on data-driven decision-making through use of run charts • Increased attention to protecting client privacy and confidentiality resulting in increased client satisfaction and trust • Peer-to-peer learning between midwives and VHTs, VHTs and VHTs • More comprehensive counseling to avoid unnecessary referrals
Service provider competence	• Changes to improve midwife-VHT mentorship and supervision • Emphasis on data-driven-decision-making through use of run charts • Monthly meetings of VHTs with midwife and mentoring • CBFP service provision assessment • Peer-to-peer learning between midwives and VHTs, VHTs and VHTs
Interpersonal relations	• Increased engagement of men and couples on family planning • Emphasis on data-driven decision-making through use of run charts • Monthly meetings of VHTs with midwife and mentoring • Focus on teamwork, shifts in responsibility to allow midwives to focus on complicated cases • Improved supervision, support, and relationships between providers for more positive attitude and morale • Increased attention to protecting client privacy and confidentiality resulting in increased client satisfaction and trust
Continuity and follow-up mechanisms	• Increased follow-up visits, calls with clients, supporting client FP disclosure to partners for improved service quality and delivery • Emphasis on data-driven decision-making through use of run charts • Facilitated discussions and learning sessions, monthly meetings
Appropriate constellation of services	• Improved linkages between VHTs and health center for referrals to long-acting and permanent methods and other health services • Integration of QIC approach in CBFP with other health service areas (immunization, maternal and child health) • Use of QIC in CBFP platform for health talks, counseling, and use beyond FP

VHT, Village Health Team; CBFP, community-based family planning; QIC, quality improvement collaborative.

This is the first process evaluation of a community-based QIC model in CBFP in Uganda. Bazos *et al.* conducted a proof-of-concept study of a microsystems QI approach to strengthen routine immunization in Uganda ^33^. While the QI approaches are similar in their emphasis on identifying local solutions, Bazos *et al.* focused their approach on the routine immunization system, which is facility-focused, with immunizations provided by clinic-based providers. Tancred *et al.* implemented a community-level QI program with village volunteers in neighboring districts in Tanzania and Uganda for maternal and child health services ^9^. The QIC described in this paper focuses on FP, with the VHTs serving as the core service delivery providers at the community level. In addition, while some health centers had established a facility QI team through previous QI projects, this program was the first to implement a QIC with VHTs at the core, extending PDSA cycles to service delivery by community-based providers. Engaging VHTs as integral players in the QIC strengthened the linkages between the health services provided in communities and those provided in health centers.

Ludwick *et al.* evaluated CHW performance in Uganda and found that factors related to supportive supervision and relationships with other health workers may be strongly associated with variances in performance outcomes ^34^. Studies have highlighted the importance of supportive supervision, supervisor legitimization of CHW roles to communities and positive interactions between CHWs and health workers and their linkages to CHW motivation and performance ^35, 36^. The QIC is an approach that may support improved overall CHW performance. With the QIC, a culture of mentorship has emerged that replaces the formal hierarchical midwife-VHT relationship, with peer-to-peer learning accelerating learning and scale-up from district to district. QICs focus on improvements based on collection and reflective analysis of data and testing of small, feasible changes, rather than a didactic pedagogical approach. Further, a QIC may change the way services are delivered. The CHWs identified change ideas that redesigned their approach to providing services to clients as described in Table 5; such as doing home visits with couples using FP with other couples to encourage FP use, using male expert clients, and encouraging women to inform male partners and come to appointments with them. Pilot sites experienced accelerated scale-up of the approach to other sites through the deliberately designed peer-to-peer knowledge and experience transfer process. At the same time, there was room for adaptation of change ideas to the new contexts, as was shared by Oyam key informants. Our process evaluation showed that there was flexibility in the QIC scale-up process – through the evolution of change packages – contributed to enhanced peer learning, ownership of the model and successful scale-up. This focus on local adaptation to each scale-up site is a unique characteristic of this QIC program as change packages and tools are often static from the first site and tend to be scaled more prescriptively. In addition, all change ideas were locally driven and culturally appropriate because they were identified by VHTs, who are closer to communities than health providers. The emphasis on trust between VHTs and clients in our findings shows that community-based QICs can be successful in being client-centered. Investing sufficient time in the pilot site helped to identify what works and continuously engaging health system leaders throughout the QI process allowed for sustainability planning during scale-up to achieve and maintain improvement gains.

Some of the challenges that were voiced by participants can be addressed by adjusting tools and terminology for VHTs and clients, being more gradual in capacity building of new teams and simplifying the change identification process. However, the challenges also show the limits of the improvement process. Availability of supplies, for example, is a systems issue that cannot be resolved at the community level or by improving service delivery. On the other hand, progress achieved in male engagement shows that a collaborative can tackle even complex sociocultural factors.

Working with CHWs on a QIC presents different challenges as compared to working at the facility level. In Uganda, VHTs are volunteers, with low levels of literacy and no previous data analysis experience. However, their enthusiasm and creativity proved important to improving processes. We also demonstrate that despite their literacy levels, VHTs given adequate support through mentorship and learning sessions are capable of implementing the PDSA concept, data analysis and continuous improvement. To strengthen the implementation of this CBFP QIC and other community-based QICs, we provide program recommendations in Box 1.

Box 1. **Program recommendations**

- Embed the quality improvement collaborative (QIC) approach in the Village Health Team (VHT) curriculum.
- Incorporate recording, plotting, and interpretation of data into VHTs’ and midwives’ training.
- Engage clients as key stakeholders in the improvement effort.
- Conduct inter-facility and inter-district joint learning sessions to increase VHTs initiative and desire to perform better and to accelerate adaptation of proven change ideas.
- Recognize the contributions of VHTs and provide them with transport stipends to enable them to submit reports regularly.
- Include budgets for community-based family planning (CBFP) and QI initiatives in FP costed implementation plans.
- Have an integrated QI approach at facility level, so that the CBFP QI efforts complement other QI projects for non-FP services.
- Consider stock availability and building the capacity of VHTs and midwives to forecast their supply needs as quality improves and demand for services increases.
- Develop an adaptable resource package based on Busia’s experience as a learning site for other implementing partners and the government to adopt or adapt for future QIC programs. The resource package should be housed in a central institution, such as the Ministry of Health’s QI coordination committee, which coordinates the national QI strategy.

Results from this process evaluation are not generalizable and do not allow a causal inference that this approach has a “proven” effect, particularly as it is largely qualitative. Selection of health centers and VHTs from Busia and Oyam districts to participate in the QIC was not random, resulting in possible selection bias. Busia district was considered a high performing CBFP district lending it to serve as the QIC learning site, but not representing an average performing district in the country. The data used in this evaluation may also be at risk of measurement error, as they were reported by individuals. However, the many lessons learned and the program description may be useful in supporting other programs interested in replication and adaptation efforts.

## Conclusions

This process evaluation shows that it is feasible to implement the QIC approach to improve service delivery by VHTs. Our data suggests that such QICs can increase uptake of FP and improve retention of clients on FP methods.

## Data availability

### Underlying data

Due to data protection issues, publicly sharing data from the qualitative interview transcripts is not feasible. While identifying names have been removed from the transcripts, based on the information provided in the interviews, it would be possible to identify the participants given the position of the respondent, their health center, and shared experiences. We also state in the IRB-approved and waived verbal consent forms that we would share results only, that only study team members would have access to the interview data, and no information identifying the participant would be shared. Additionally, we do not explicitly mention sharing the underlying data from the interviews. While intermediary transcript data maybe not be able to be fully de-identified without compromising anonymity or the quality of the transcripts, depending on the purpose of the request (for example, a request by researchers or implementers of community-based QI for the purpose of informing QI program scale-up), we may be able to discuss the access and use of the qualitative data, if approved by all relevant parties (IRB, program staff, and research staff). This would be done on a case by case basis – the corresponding author can be contacted for initiating discussions on data access and use.

Zenodo: Program data - quality improvement for CBFP. http://doi.org/10.5281/zenodo.2647624 ^29^

This project contains the following underlying data:

- programdatabyhealthcenter_QIC.csv (spreadsheet containing de-identified program data)

Data are available under the terms of the Creative Commons Attribution 4.0 International license (CC-BY 4.0).

### Extended data

Zenodo: In-depth Interview and Focus Group Discussion Guides. http://doi.org/10.5281/zenodo.2647629 ^22^

This project contains the following extended data:

- 1_QI_Interview guides_VHTs Busia.docx
- 2_QI_Interview guides_VHTs Oyam.docx
- 3_QI_Interview guides_Midwives Busia.docx
- 4_QI_Interview guides_Midwives Oyam.docx
- 5_QI_Interview guides_Incharges Busia.docx
- 6_QI_Interview guides_Incharges Oyam.docx
- 7_QI_Interview guides_DHO Busia.docx
- 8_QI_Interview guides_DHO Oyam.docx
- 9_QI_Interview guides_MOH representative.docx
- 11_QI_Interview guides_MaleClients Busia.docx
- 12_QI_Interview guides_FemaleClients Busia.docx
- 13_QI_Interview guides_CouplesClients Busia.docx

Data are available under the terms of the Creative Commons Attribution 4.0 International license (CC-BY 4.0).
