# Supplementary material for: A process evaluation of the quality improvement collaborative for a community-based family planning learning site in Uganda
Source: Gates Open Res. 2019 Aug 29;3:1481. Originally published 2019 Jun 4. [Version 2] doi: 10.12688/gatesopenres.12973.2 (PMC6650767; doi:10.12688/gatesopenres.12973.2)
Supplement: Peer review history

# Peer review: Reviewer response for version 1

**DOI:** 10.21956/gatesopenres.14080.r27478

**Published:** 2019-07-22

**Authors:** Mirwais Rahimzai

**Affiliations:** 1 University Research Co., LLC, Kampala, Uganda

I think it is an excellent article. These are my major comments:

- The article nicely captures details on how quality improvement methology contribute to improving services and uptake of FP methods.
- To make it more reader friendly, some details on "HOW" QI improved care would be helpful. This will be a few lines explaining how the processes of care were redesigned.
- Some more information on how lessons learned were taken to scale will help.

I confirm that I have read this submission and believe that I have an appropriate level of expertise to confirm that it is of an acceptable scientific standard.

# Response

**Published:** 2019-08-14

**Authors:** Christine Kim

Thank you for your review and helpful comments on our manuscript. Your suggestions to add some content in the discussion on how services were redesigned using the QI approach and additional lessons from scale-up have been incorporated.

# Peer review: Reviewer response for version 1

**DOI:** 10.21956/gatesopenres.14080.r27404

**Published:** 2019-07-05

**Authors:** Claudia Hanson

**Affiliations:** 1 Department of Public Health Sciences, Karolinska Institutet, Solna, Sweden

The paper describes a family planning project that uses the collaborative model to improve the provision of family planning methods in Uganda. The intervention is innovative in the way that it links village health teams and health facilities. Several improvement ideas were developed jointly within these two groups. Also, data were not only collected at the facility level but by communities. Communities described data collection as empowering.

 The methods used to evaluate are adequate in the sense that the authors aim to describe what they saw to happen in relation to two key indicators on FP-follow-up visits and new couples. The methods chosen do not allow to make a causal inference that this approach has a “proven” effect, and I believe the conclusion are carefully enough formulated that this limitation is clear. It could however, be put more clearly at the end of the discussion section.

I confirm that I have read this submission and believe that I have an appropriate level of expertise to confirm that it is of an acceptable scientific standard.

# Response

**Published:** 2019-08-14

**Authors:** Christine Kim

Thank you for your thoughtful review of our manuscript. As you mentioned, it is very important to clearly state the level of inference that can be made from our study. We have clarified this in our limitations paragraph in the discussion as you have suggested.
